# Wide QRS complex tachycardia after surgical repair of an isolated atrial septal defect: What is the mechanism?

**DOI:** 10.1002/joa3.12487

**Published:** 2021-02-22

**Authors:** Norman C. Wang

**Affiliations:** ^1^ Division of Cardiology Department of Medicine University of Pittsburgh School of Medicine Pittsburgh PA USA

**Keywords:** atrial septal defect, catheter ablation, wide QRS complex tachycardia

## Abstract

Macroreentrant atrial tachycardia within the right atrium is the dominant mechanism in patients with prior surgical repair of atrial septal defects, with dual‐loop circuits much more common than single‐loop circuits. This case highlights the importance of clinical history for predicting arrhythmia mechanisms. Considering prior cardiac surgery may assist in preprocedural preparations and discussions regarding potential risks and benefits of catheter ablation.

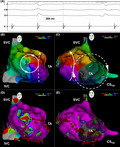

## 1 CASE SUMMARY

**FIGURE 1:**
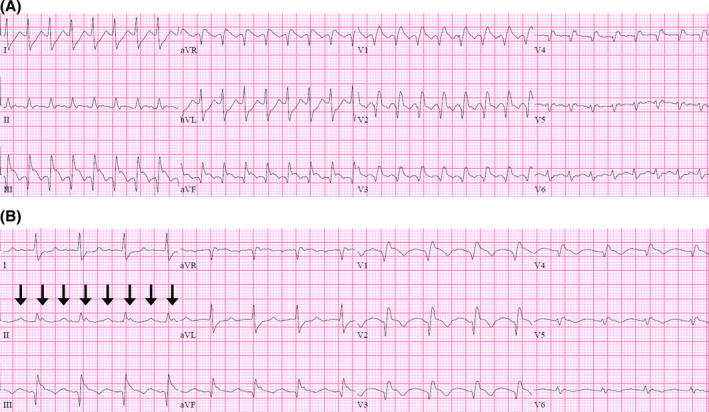
(A) Initial electrocardiogram. (B) Electrocardiogram after amiodarone revealed inferiorly directed P‐waves (arrows)

A 44‐year‐old man with prior surgical repair of an isolated atrial septal defect (ASD) at 17 years of age presented with palpitations. The electrocardiogram demonstrated a wide QRS complex tachycardia at 196 beats per minute (bpm) with a right bundle branch block of 136 ms (Figure 1A). Intravenous adenosine administered in three separate doses of 6, 12, and 12 mg had no effect. An intravenous amiodarone bolus of 150 mg caused a decrease in heart rate to 97 bpm (Figure 1B). What is the mechanism?

## 2 COMMENTARY

The differential diagnosis of the wide QRS complex tachycardia included supraventricular tachycardia with aberrant ventricular conduction and ventricular tachycardia. There were no features highly suggestive of ventricular tachycardia. Although a full discussion is beyond the scope of this manuscript, a comprehensive review by Verecki is available.^1^ Halving of the heart rate after amiodarone revealed P‐waves, confirming supraventricular tachycardia with 2:1 atrioventricular conduction. The inferiorly directed P‐wave axis suggested atrial tachycardia.

Houck et al. reported supraventricular tachycardia occurrence in 44 patients after a median of 16 years (interquartile range, 4‐28 years) after ASD repair. They were screened from 245 patients with surgical ASD repair.^2^ Macroreentrant atrial tachycardia (MRAT) within the right atrium is the dominant mechanism in this population with dual‐loop circuits much more common than single‐loop circuits.^3^ Therefore, the most likely diagnosis was dual‐loop MRAT.

**FIGURE 2:**
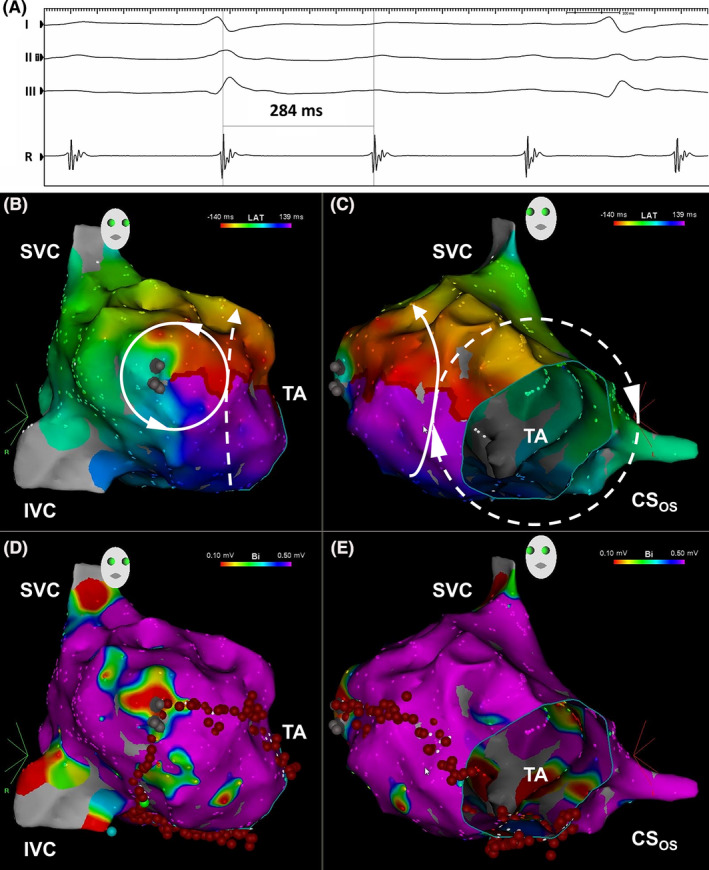
(A) Surface leads I, II, and III with intracardiac reference in the mid‐coronary sinus. Activation maps in the right anterior oblique (B) and left anterior oblique (C) projections. Voltage maps in the right anterior oblique (D) and left anterior oblique (E) projections. Areas with positive contact but no electrograms corresponded to the anticipated location of the atriotomy scar (gray dots). Ablation lesions (red dots) and the lesion associated with arrhythmia termination (green dot) are marked. See text for details. CS_OS_, coronary sinus ostium; IVC, inferior vena cava; SVC, superior vena cava; TA, tricuspid annulus

Electrophysiology study was performed. A preprocedural transesophageal echocardiogram estimated a left ventricular ejection fraction of 35%‐40%. Local electrograms demonstrated a proximal to distal activation within the coronary sinus and a clockwise pattern around the tricuspid annulus (not shown). The atrial cycle length was 284 ms (Figure 2A). The postpacing interval minus tachycardia cycle length was 25 ms from the proximal coronary sinus and 10 ms from the low lateral right atrium, confirming typical clockwise atrial flutter dependent on the cavotricuspid isthmus.

Activation and voltage maps were created in a point‐by‐point fashion with the CARTO® 3 electroanatomic mapping system and an irrigated 3.5 mm tip THERMOCOOL SMARTTOUCH® radiofrequency ablation catheter (Biosense Webster). For activation mapping, a window of interest was set to identify the mid‐diastolic isthmus as previously described.^4^ A color‐coded activation sequence (red → orange → yellow → green → cyan → blue → purple) corresponded to chronology within each atrial cycle, with the mid‐diastolic isthmus represented by the interface between red and purple, or “early‐meets‐late.” Areas with no signal, but positive contact, were labeled with gray dots and corresponded to the anticipated location of the atriotomy scar.

Figure 2B, a right anterior oblique projection, demonstrates a counterclockwise loop around the atriotomy scar (solid circle) and part of the tricuspid annulus loop (dashed line with arrowhead). Figure 2C, a left anterior oblique projection, demonstrates a clockwise loop around the tricuspid annulus (dashed circle) and part of the atriotomy loop (solid line with arrowhead). Dual‐loop reentry has been defined when activation within each loop spans ≥90% of the atrial cycle length.^4^ Both loops met criteria for MRAT as 279 ms of the 284 ms cycle length (98%) was mapped and “early‐meets‐late” was observed. Dual‐loop MRAT after surgical ASD repair, first described by Shah et al.,^5^ was confirmed.

Voltage maps in the right anterior oblique and left anterior oblique projections are shown in Figures 2D and E, respectively. Signal amplitude thresholds were set as ≤0.1 mV for dense scar (red) and ≥0.5 mV for healthy tissue (purple). Amplitudes in between were considered border zone. MRATs may also occur around scars from prior cannulation, atrial septal patches, the coronary sinus ostium, and other regions of block.^3^ There were no cannulation scars observed.

Radiofrequency ablation of the cavotricuspid isthmus, from the 6 o'clock position of the tricuspid annulus to the inferior vena cava, was performed. A second line was delivered from the atriotomy scar to the 9 o'clock position of the tricuspid annulus without effect. This “common corridor” is an attractive target, but catheter instability, as experienced here, is a reported shortcoming.^5^ A third line was delivered from the atriotomy scar to the inferior vena cava, leading to arrhythmia termination. In sinus rhythm, the patient had a right bundle branch block of 140 ms. The patient had no palpitations or arrhythmias after 4 years of follow‐up.

This case highlights the importance of clinical history for predicting arrhythmia mechanisms. Considering prior cardiac surgery may assist in preprocedural preparations and discussions regarding potential risks and benefits of catheter ablation. Magnin‐Poull et al. described termination or arrhythmia transformation using conventional entrainment mapping in three of four patients with MRAT and prior surgical ASD repair.^3^ Therefore, electroanatomic mapping should be considered in these clinical scenarios as a first‐line mapping strategy.

## CONFLICT OF INTEREST

Author declared no conflict of interest for this article.
